# A novel rabbit fixator made of a thermoplastic mask for awake imaging experiments

**DOI:** 10.1038/s41598-021-81358-6

**Published:** 2021-01-15

**Authors:** Rencai Lu, Li Hou, Siyu Wang, Bo She, Hong He, Wentao Gao, Sidang Wang, Dongdong Xv, Yunhai Ji, Shasha Yang, Zhaohui Yang, Shaobo Wang

**Affiliations:** 1 PET-CT Center, the First People’s Hospital of Yunnan Province, Kunming, 650032 Yunnan Province China; 2 Department of Radiation, the First People’s Hospital of Yunnan Province, Kunming, 650032 Yunnan Province China; 3 Yunnan Key Laboratory of Primate Biomedical Research, Institute of Primate Translational Medicine, Kunming University of Science and Technology, Kunming, 650093 Yunnan Province China

**Keywords:** Animal physiology, Zoology, Medical research

## Abstract

This study aimed to develop and validate a novel rabbit fixator made from a thermoplastic mask for awake imaging experiments. When heated in a hot-water bath at 65–70 °C for 2–5 min, the thermoplastic mask became soft and could be molded to fit over the entire body of an anesthetized rabbit (4 ml of 3% pentobarbital sodium solution by intramuscular injection). Twenty rabbits were randomly divided into fixator (n = 10) and anesthesia (n = 10) groups. The animals’ vital signs, stress hormones (cortisol and adrenaline)**,** and subjective image quality scores for the computed tomography (CT), positron emission tomography (PET), and magnetic resonance imaging (MRI) scanning were measured and compared. Phantom CT, MRI and PET studies were performed to assess the performance with and without the thermoplastic mask by using image agents at different concentrations or with different radioactivity. The respiration rate (RR), systolic blood pressure (SBP), diastolic blood pressure (DBP), peripheral capillary oxygen saturation (SpO_2_) and body temperature (T) decreased after anesthesia (all *P* < 0.05) but did not significantly decrease after fixation (all *P* > 0.05). The heart rate (HR), cortisol and adrenaline did not significantly decrease after either anesthesia or fixation (all *P* > 0.05). The subjective image quality scores for the CT and MRI images of the head, thorax, liver, kidney, intestines and pelvis and the subjective image quality scores for the PET images did not significantly differ between the two groups (all *P* > 0.05). For all examined organs except the muscle, ^18^F-FDG metabolism was lower after fixation than after anesthesia, and was almost identical of liver between two groups. The phantom study showed that the CT values, standard uptake values and MR T2 signal values did not differ significantly with or without the mask (all *P* > 0.05). A novel rabbit fixator created using a thermoplastic mask could be used to obtain high-quality images for different imaging modalities in an awake and near-physiological state.

## Introduction

Rabbits are one of the most used experimental animals in biology and medical research because they are docile, easy to raise, moderate in size and omnivorous. For disease modeling in rabbits, various imaging procedures, such as computed tomography (CT), positron emission tomography (PET), magnetic resonance imaging (MRI) and single-photon-emission CT (SPECT), are typically required^1–4^. Imaging research on rabbits often requires the animal to be restrained in a manner that does not affect the image quality or the animal’s physiological status.

Anesthesia is a commonly used approach that can ensure fixation of an experimental rabbit for a long period and allows for the acquisition of relatively stable images. However, anesthesia affects the respiration, blood pressure, and heart rate of the experimental animals^5,6^. It can lead to apoptosis of rabbit tissue cells^7^, making it particularly difficult for histopathological studies to control for variables. The differences in the tolerance levels of different individual rabbits to anesthesia, awakening during the extended examination procedure, and postural changes in multiple scans may affect the imaging results. Additionally, substantial reductions in the animal’s metabolic rate are observed in rabbits under anesthesia^8^.

Therefore, an appropriate rabbit fixator is an alternative to anesthesia for performing relevant inspections, operations, and experiments. However, the metal accessories in current fixators lead to artifacts in CT, limit their use for MRI and interfere with photon acquisition during PET and SPECT scanning. Additionally, the fixation is not tight enough, leading unsatisfactory imaging results. A novel restraining device made of transparent acrylic material, developed by Barbosa CH et al.^9^, allows the easy placement of animals in different body positions and can yield high-quality images for rabbit imaging experiments, but it only works after the animal has been anesthetized, otherwise, the rabbit will struggle and capturing motion in the artifact is unavoidable. Thermoplastic masks are plastic mesh sheets that become soft upon heating and can be molded to any shape, which they hold when cooled. The thermoplastic mask has an excellent fixation effect in positioning during radiotherapy and results in high-quality imaging^10–12^.

Therefore, this study aimed to develop and validate a new rabbit fixator made of a thermoplastic mask to use for imaging experiments, which provides anesthesia-free fixation, little effects on vital signs, the high-quality imaging for multiple imaging modalities, and long-term examination in the same body position.

## Materials and methods

### Ethical approval

The study was conducted in strict accordance with the recommendations of the Kunming Medical University for the Care and Use of Laboratory Animals. The protocol has been approved by the Animal Experiment Ethics Committee of Kunming Medical University (Permit Number: KMMU2019091), and followed the reduce, refine, and recycle principle for experimental animals. All experiments were performed in accordance with the relevant guidelines and regulations. All methods were carried out in compliance with the ARRIVE guidelines. A patent has been authorized by the National Intellectual Property Administration of China under number ZL 2019 2 0,133,870.4.

### Experimental animals

Twenty healthy female rabbits weighing 2.1–3.0 kg were provided by the experimental animal center of Kunming Medical University and were randomly divided into the fixator group (n = 10) and the anesthesia group (n = 10), using a computer based random order generator. The rabbits were two months old and had body weights of 2.4 ± 0.3 kg and 2.4 ± 0.4 kg, respectively (mean ± SD). We selected a small sample size because the novel rabbit fixator was evaluated for the first time in the present study, and therefore, the initial intention was to gather basic evidence regarding the use of this novel rabbit fixator made of thermoplastic mask in awake imaging experiments.

### Fixator

#### Structure and materials

The fixator was made from a thermoplastic mask (Klarity Medical Products, Guangzhou, China) and strapping tape. The thermoplastic mask had a size of 2609.6 cm^2^ (56.0 cm × 46.6 cm) and a thickness of 0.24 cm; it was made of a specially synthesized polymer polyester and low-temperature thermoplastic plate cut into sheets and frames or edges.

#### Preparation

**Figure 1:**
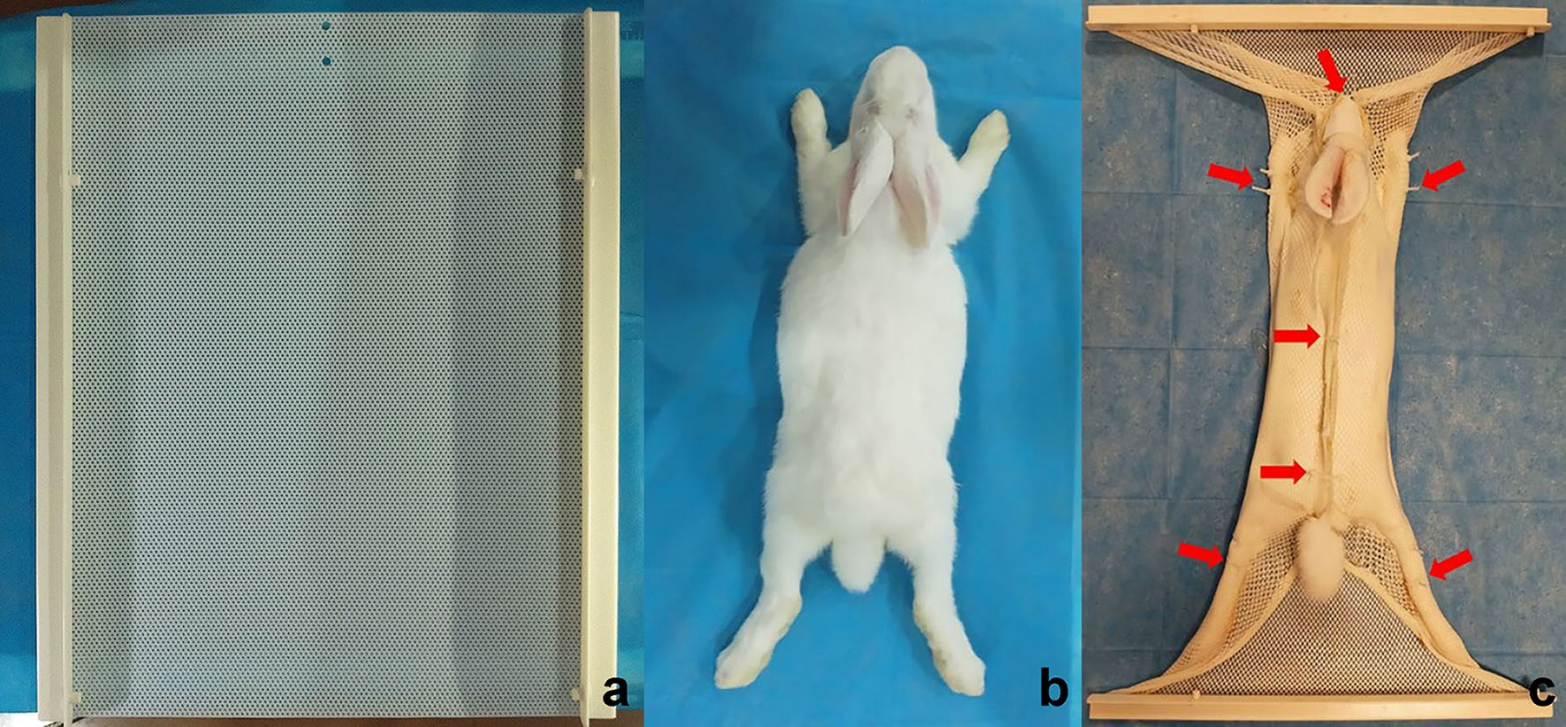
**(a)**: The overall shape of the thermoplastic mask before thermoplastic molding. **(b)**: The rabbit’s body position. **(c)**: The thermoplastic mask covered the entirety of the rabbit’s body, including its head and all of its limbs; the red arrows indicate the strapping tape positions.

(1) An experimental rabbit with a median body weight (2.5 kg) was anesthetized by an intramuscular injection of 4 ml 3% pentobarbital sodium for subsequent use. (2) The thermoplastic mask was softened via immersion in a hot-water bath (MED-TEC, INC, PO Box 320 Orange city, IA 51,041 USA) at 65–70 °C for 2–5 min and was then removed from the water bath and wiped dry. (3) The thermoplastic mask was placed on a flat platform after being slightly stretched. (4) The anesthetized rabbit was stretched and placed in the prone position on top of the mask. The thermoplastic mask was rapidly folded from both edges to the center such that it covered the entire surface of the rabbit and was molded to fit the rabbit’s contours. (5) The mask completely hardened after 10–20 min and could be used as the fixate mold around a rabbit in the prone position. (6) If the fixator was not satisfactory, the rabbit was removed, and then the mask was softened in the water bath again and reshaped by repeating steps (2) to (5). (7) Once the fixator was completed, it could be used to restrain the rabbit without anesthesia by placing the rabbit into the mold and fixing it with eleven pieces of strapping tape (Fig. 1). After the experiment was completed, the strapping tape was cut and the rabbit was removed from the fixator.

#### Examination of the fixator

The preparation time, the changes in the rabbit’s vital signs and serum stress hormones (cortisol and adrenaline) before and after restraining or anesthesia were evaluated and compared. The preparation time was defined for fixator group as the beginning of restraining until the respiration and heart rate stabilized, and for anesthesia group, it was defined as the time it took for the muscle tension and corneal reflex to completely disappear after injecting the anesthetic agent. The SurgiVet multiparameter vital sign monitor (Smiths Medical ASD, INC, St Paul, MN 55,112 USA) was used for monitoring the rabbit, including the blood pressure (Systolic blood pressure, SBP; Diastolic blood pressure, DBP), respiration rate (RR), peripheral capillary oxygen saturation (SpO_2_), heart rate (HR), and body temperature (T). When the rabbits were completely restrained in the fixator or anesthetized, the animal’s blood pressure was measured by hooking a cuff to the rabbit’s limb; the cuff inflated and gave a reading automatically. The SpO_2_ and HR values were measured by a sensor attached to the pinna. The body temperature was measured by an infrared thermometer and the RR was calculated by counting.

Blood serum samples were harvested immediately and stored at –20 C in labeled EDTA tubes before and after restraining or anesthetizing. When all the samples had been collected, their cortisol and adrenaline concentrations were evaluated at a KingMed’s laboratory facilities using ELISA methodology (cortisol) and radioimmunoassay (adrenaline) (KingMed Diagnostics Group Co., Ltd, Guangzhou, China).

### Imaging devices

After restraining, the rabbits were subjected to PET, CT, and MRI examinations. A Philips Ingenuity TF PET/CT scanner equipped with 64-slice spiral CT capability was used for PET/CT scanning. The image resolution of the PET scanner was 4.7 mm FWHM (Full Width Half Maximum) at a distance of 1 cm from the center, and that of the CT scanner was 24 LP/cm. Philips IntelliSpace Portal v7.0.4.20175 was used for postprocessing. This system allowed the simultaneous acquisition of CT, PET, and PET/CT fusion images of the rabbit. ^18^F-FDG was produced through the F300e ^18^F-FDG synthesis module by the Sumitomo HM-10HC cyclotron. ^18^F-FDG is the most widely used radiotracer for PET-CT, and it is a radiolabeled glucose analog in which the positron emitter radioactive isotope ^18^F replaces the hydroxyl group at the C2 position in the glucose molecule; ^18^F-FDG is phosphorylated by hexokinase II to ^18^F-FDG-6-phosphate and accumulates in metabolically active cells^13^.

The Siemens Prisma 3.0-T MRI scanner was used for MRI scanning with abdominal coils covering the whole body of the rabbit; for the fixator group, the rabbits’ ears were plugged with earplugs during MRI scanning.

### Examinations

The rabbits from both groups were dry-fasted for 12 h. Ten rabbits were restrained with the novel fixator, and the other ten rabbits were anesthetized with 4 ml of 3% pentobarbital sodium solution by intramuscular injection.

Next, ^18^F-FDG was injected at a dose of 38.85–45.88 MBq into the rabbits' ear-rim auricular vein. The rabbits were transferred to the PET/CT scanning room. Static images were acquired at 30 min, 60 min, 90 min, and 120 min from head to tail in the prone position. The tube voltage was 140 kV and tube current was 300 mAs for CT scanning. The iDose technique was used with a field of view (FOV) of 600.0 mm, a matrix of 512 × 512, a pitch of 0.83 and a reconstruction slice thickness of 1 mm.

CT images were also used to produce the attenuation correction value for the PET emission reconstruction, as well as for the anatomic localization of the ^18^F-FDG uptake. PET scanning included seven beds with a slice thickness of 1.0 mm and an image matrix of 128 × 128. Dual-phase CT scans were performed after the PET scans to check for motion. The scans took 1 min per bed position for a total of approximately 10 min. During the entire scanning process, the rabbits were continuously restrained or anesthetized until the last scanning procedure was complete.

Once the image acquisition was completed, the images from each group were opened at the workstation. Then, the standard uptake values (SUVs) of the brain, thorax, liver, kidney and muscle were measured by drawing a region of interest (ROI) of 15 pixels, and the ^18^F-FDG metabolism values of the two groups were compared.

The coronal sagittal T2-weighted image (T2 WI) and diffusion weighted image (DWI) were acquired during MRI scanning, and the half Fourier single shot turbo-spin echo (HASTE) sequence was used in the MRI T2 WI acquisition. The parameters for T2 WI were as follows: TR 1,200.0 ms, TE 93 ms, matrix 256 × 256, FOV 500.00 mm, slice thickness 4 mm, and slice interval 5 mm. The image resolution of the T2 WI image was 384 for the frequency encoding direction and 268 for the phase encoding direction. An echoplanar imaging (EPI) sequence was used in the MRI DWI acquisition with a b value of 50 and 500 s/mm^2^. The parameters for DWI were as follows: TR 9,800.0 ms, TE 49 ms, FOV 280.00 mm, slice thickness 5 mm; the image resolution of the DWI image was 164 for the frequency encoding direction and 164 for the phase encoding direction. The images were opened at the workstation after scanning.

### Subjective image quality

The subjective image quality was evaluated by two independent radiologists (Bo She and Shaobo Wang), each with more than 10 years of experience in radiology diagnosis. The subjective image quality was assessed in five different body regions, which also corresponded to typical CT and MRI examination regions, i.e., the head, neck, thorax, abdomen and pelvis. A five-point Likert scale was used by the reviewers to rate the overall image quality as follows^14–16^: 1, Nondiagnostic, critical structures completely obscured with severely impaired readability; 2, Poor, notable artifacts with moderately impaired image readability; 3, Moderate, moderate artifacts with mildly impaired readability; 4, Good, artifact present, but no significant impairment; 5, Excellent, unimpaired readability, no artifact with high confidence in diagnosis.

For quality assessment of the PET images, the images were windowed at each interpreter’s preference. Each interpreter then graded the image quality subjectively as good (3), moderate (2), poor (1), or nondiagnostic (0). In general, the smoothness versus graininess of the liver was used as a criterion to distinguish poor from moderate quality. The image sharpness, which is usually observed best in the thorax at the lung/chest wall junction, was used to distinguish good from moderate quality^17,18^.

### Phantom study

We performed phantom studies to assess the reliability of the rabbit fixator made from a thermoplastic mask. Therefore, a dedicated phantom was constructed; it was a cuboid that contained six tubes with a diameter of 10 mm. Six tubes were filled with ioversol (Hengrui Medicine Co., Ltd, Jiangsu, China) at different concentrations (0%, 10%, 20%, 30%, 40%, 50%) in distilled water for CT scanning, ^18^F-FDG with different radioactivity values (0 MBq, 3.7 MBq, 7.4 MBq, 11.1 MBq, 14.8 MBq, 18.5 MBq) for PET scanning, and gadolinium-diethylene triamine pentaacetic acid (Gd-DTPA) (Consun Medicine Co., Ltd, Guangzhou, China) at different concentrations (0%, 10%, 20%, 30%, 40%, 50%) for MRI scanning. All the scanning parameters were the same as above, and all datasets were transferred to the postprocess workstation. The CT, SUV and MR T2 signal values were measured by drawing an appropriate ROI that contained 10 pixels for each value measurement. The ROIs were placed in the central region along the tube axis congruently in all reconstructed images. The resulting CT SUV or MR signal values at the different concentrations or different radioactivity values were calculated as the mean value over all six ROIs.

### Statistical analysis

Statistical analysis was performed with the software R GUI 4.0.2 (The R foundation for statistical computing, The United States). The graphs were plotted with GraphPad Prism 8.0. Data are expressed as the mean ± SD ($\stackrel{-}{x}$±s). The continuous variables, such as the vital signs and subjective image quality scores, were analyzed with a permutation test. For quantitative comparison of the phantom study with and without the thermoplastic mask, the CT, SUV and MR T2 signal values were compared using Bland–Altman plots in differences with limits of agreement ± 1.96 standard deviations. Equivalence was defined by the inclusion of the line of equality in the 95% confidence interval (CI) of the mean difference. *P* < 0.05 was statistically significant.

## Results

### Comparison of vital signs and stress hormones before and after fixation or anesthesia

The body weights of the rabbits in the fixation and anesthesia groups did not differ significantly (*P* = 0.796). The preparation time for the fixator group was significantly shorter than that for the anesthesia group (9.3 ± 2.7 min *vs.* 13.7 ± 2.9 min; *P* = 0.004). The vital signs and stress hormones of the rabbits from the fixator group before fixation did not differ significantly from that after fixation (all *P* > 0.05). For the rabbits from the anesthesia group, the respiration, blood pressure, SPO_2_ and body temperature values were significantly lower after anesthesia (RR: 59.7 ± 4.2breaths/min *vs.* 45.5 ± 11.8 breaths/min; SBP: 106.7 ± 15.3 mmHg *vs.* 97.9 ± 23.8 mmHg; DBP: 79.3 ± 19.2 mmHg *vs.* 54.1 ± 19.1 mmHg; SpO_2_: 89.7 ± 0.6% *vs.* 84.5 ± 5.5%; T: 38.6 ± 0.5 ℃ *vs.* 36.2 ± 0.6 ℃; all *P* < 0.05) but the stress hormone values did not differ significantly (both *P* > 0.05) (Table 1).

**Table 1:** Vital sign and stress hormones changes of the two groups ($\stackrel{-}{{\varvec{x}}}$±*s*).

Item	Fixator group (n = 10)		*P*	Anesthesia group (n = 10)		*P*
	Before fixation	After fixation		Before anesthesia	After anesthesia	
Body weight (kg)	2.4 ± 0.3		–	2.4 ± 0.4^a^		–
Preparation time (min)	9.3 ± 2.7		–	13.7 ± 3.0^b^		–
RR (breaths/min)	64.6 ± 7.0	65.5 ± 5.1	0.944	59.7 ± 4.2	45.5 ± 11.8	0.017
HR (bpm)	217.4 ± 22.4	219.4 ± 24.3	0.944	236.3 ± 8.5	226.5 ± 47.7	0.484
SBP (mmHg)	143.5 ± 33.2	137.9 ± 28.9	0.161	106.7 ± 15.3	97.9 ± 23.8	0.036
DBP (mmHg)	93.8 ± 35.0	65.8 ± 15.7	0.093	79.3 ± 19.2	54.1 ± 19.0	0.036
SPO_2_ (%)	91.8 ± 4.1	90.8 ± 4.6	0.808	89.7 ± 0.6	84.5 ± 5.5	0.025
T (℃)	39.8 ± 0.8	38.6 ± 1.1	0.107	38.6 ± 0.5	36.2 ± 0.6	0.012
Cortisol (μg/dL)	0.7 ± 0.2	1.5 ± 0.9	0.068	0.3 ± 0.1	0.6 ± 0.5	0.465
Adrenaline (pg/mL)	142.7 ± 114.3	358.7 ± 447.3	0.144	57.1 ± 8.1	358.1 ± 453.9	0.109

RR: Respiration rate; HR: Heart rate; SBP: Systolic blood pressure; DBP: Diastolic blood pressure; T: Body temperature.

^a^Compared with the fixator group, *P* = 0.976.

^b^Compared with the fixator group, *P* = 0.004.

### ^18^F-FDG metabolic parameters

**Figure 2:**
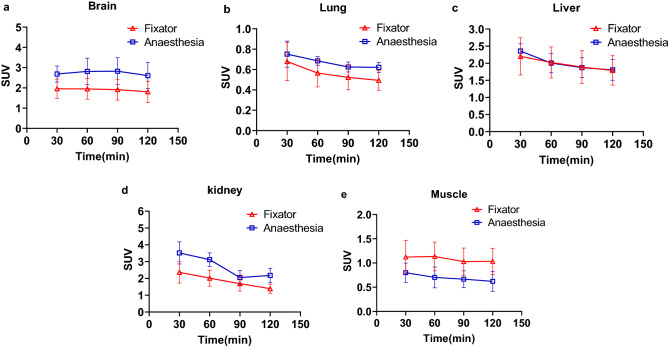
The SUV change curve of the different organs in the two groups at different time points, which shows that the SUVs of the brain **(a)**, lung **(b)**, liver **(c)**, kidney **(d)**, and muscle **(e)** gradually fell over time, and the reduction trends in the fixator group and the anesthesia group were similar.

The rabbits in both groups were subjected to a static scan at 30 min, 60 min, 90 min, and 120 min. Muscle tension recovered during PET scanning for the rabbits in the anesthesia group, and these rabbits were treated with an additional 1 ml of 3% pentobarbital sodium. The SUVs of the important organs of the rabbits in the two groups at each time point were recorded, and the results showed that data from both groups reflected the ^18^F-FDG metabolic changes in each important organ. For all examined organs except the muscle, ^18^F-FDG metabolism was lower in the fixator group than in the anesthesia group, SUV values of liver between two groups were almost identical (Fig. 2).

### Comparison of the subjective image quality scores of the CT, PET and MRI images

**Figure 3:**
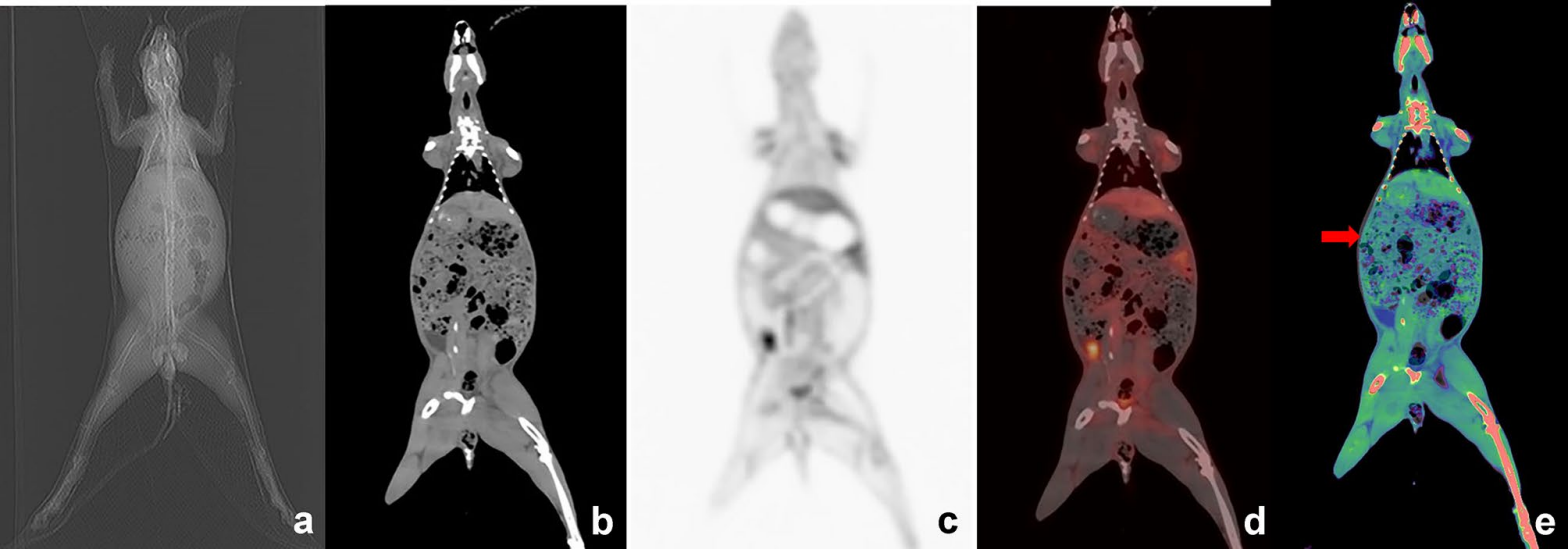
PET-CT scan for a rabbit restrained by the novel fixator. **(a)**: The CT localization image showed the overall morphology of the fixator and the rabbit under the X-ray; the rabbit cannot move when it is restrained. **(b)**: The coronal CT scan indicated that each organ of the rabbit showed well, with no metal or motion artifacts. **(c)**: The PET image showed the ^18^F-FDG images of all the organs of the rabbit that was restrained by the fixator (time point: 30 min). **(d)**: The PET/CT fusion image had perfect image quality. **(e)**: The fusion image of the dual-phase CT(before and after PET scanning (Pseudo color)) showed that the dual-phase CT images matched well except for a slight ghosting of the abdomen (red arrow).

**Figure 4:**
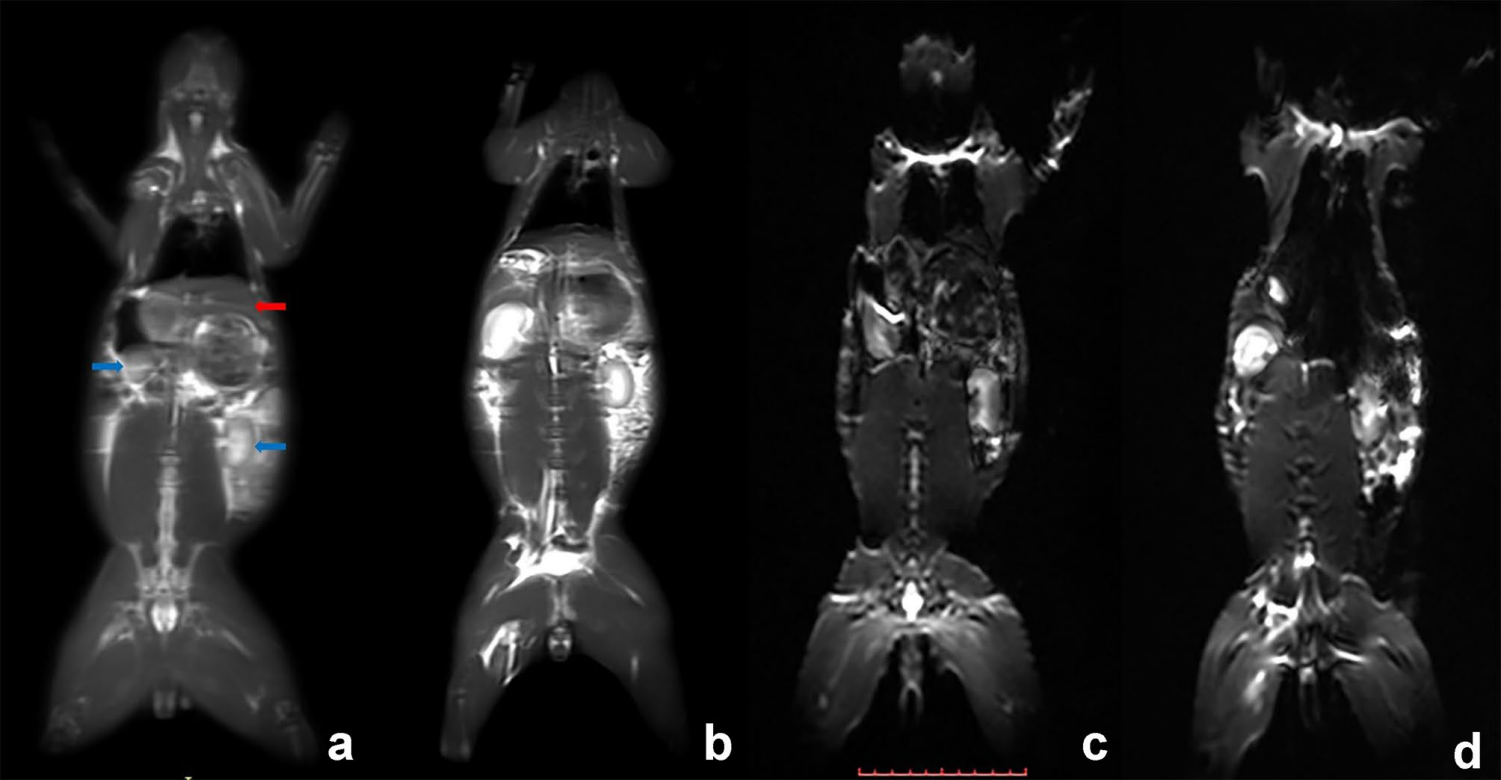
**(a,b)** The MRI T2WI image of the rabbit restrained by the fixator **(a)** or under anesthesia **(b)**. It clearly shows the main anatomical structure. such as the liver (red arrow) and kidney (blue arrow). **(c,d)** DWI image using EPI sequence for the rabbit with the fixator **(c)** or under anesthesia **(d).**

The subjective image quality scores for the CT, PET and MRI images of the head, neck, thorax, abdomen, liver, kidney, intestines, and pelvis were measured for the two groups. The results showed that the subjective image quality scores of each important organ of the two groups did not differ significantly from each other (*P* > 0.05) (Table 2, Figs. 3–4).

**Table 2:** Comparison of the subjective image quality of the CT, MRI and PET images of the two groups ($\stackrel{-}{{\varvec{x}}}$±*s*).

Region	Fixator	Anesthesia	*P*
CT			
Head	4.60 ± 0.94	4.33 ± 0.79	0.542
Neck	4.70 ± 0.79	4.33 ± 0.75	0.392
Thorax	3.70 ± 0.67	4.00 ± 0.25	0.330
Liver	4.30 ± 0.71	4.61 ± 0.33	0.379
Kidney	4.15 ± 0.97	4.39 ± 0.42	0.590
Intestines	3.20 ± 0.67	3.33 ± 0.35	0.746
Pelvis	4.00 ± 0.78	3.89 ± 0.33	0.761
MRI			
Head	4.78 ± 0.51	4.63 ± 0.44	0.631
Neck	4.61 ± 0.49	4.68 ± 0.37	0.794
Thorax	3.39 ± 0.33	3.25 ± 0.53	0.587
Liver	4.33 ± 0.35	4.19 ± 0.65	0.651
Kidney	4.61 ± 0.42	4.56 ± 0.50	1.000
Intestines	3.78 ± 0.57	3.75 ± 0.53	1.000
Pelvis	4.11 ± 0.33	3.88 ± 0.23	0.234
PET	3.50 ± 0.78	3.33 ± 0.50	0.636

### Phantom study

**Figure 5:**
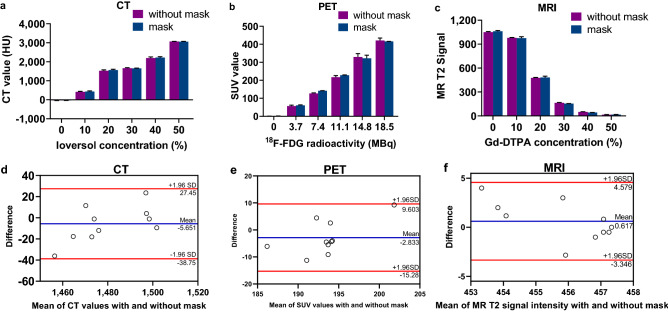
Phantom CT, MRI and PET studies with and without the thermoplastic mask. **(a–c)**: The CT, SUV and MR signal values acquired with different concentrations of ioversol, different radioactivity values or different concentrations of the Gd-DTPA agent. **(d–f)**: Bland–Altman plot of the CT, SUV or MR signal values from the phantom measurements from the images with and without the mask. The solid blue line indicates the mean difference; the upper and lower red lines indicate ± 1.96 × standard deviation.

The results of the phantom study are shown in Fig. 5. Overall, the CT, SUV or MR signal values with and without a mask did not differ significantly (CT value: arithmetic mean − 5.651, 95%CI 17.73 to 6.43, *P* = 0.318 in the Bland–Altman plot; SUV value: arithmetic mean − 2.838, 95%CI 7.38 to 1.70, *P* = 0.190 in the Bland–Altman plot; MR T2 signal: arithmetic mean 0.617, 95%CI 0.83 to 2.06, *P* = 0.359 in the Bland–Altman plot).

## Discussion

This preliminary study introduced a novel rabbit fixator made of a thermoplastic mask for imaging experiments. The thermoplastic mask is constructed of a synthetic material that shares characteristics of plastic and rubber materials. It has excellent compliance, reproducibility and conformability and can harden in 3–5 min at room temperature. Once hardened, it is lightweight, does not shrink easily, and retains its shape and size; moreover, it is strong, breathable, waterproof, completely transparent to X-rays, nontoxic, odorless, nonirritating to the skin, and biodegradable^19–21^.

Additionally, the fixator can be flexibly adjusted and reused for different rabbits with body weights within a certain range. Its use significantly decreased the preparation time, thus substantially shortening the duration of the experiment and reducing the rabbit’s suffering, as well as experimental accidents. With use of the fixator, the rabbit can be held in the same body position for image scanning in the awake state, and the rabbit's ears can be exposed for imaging tracer administration.

This study first explored how rabbits’ vital signs and stress hormones were impacted after fixation with the fixator, and the fixator showed excellent performance in the CT, PET, and MRI imaging examinations.

The results showed that the rabbits’ respiration, heart rate, blood pressure, body temperature values and stress hormones did not significantly change after fixation. Conversely, although the heart rate and stress hormones were not significantly altered, the respiration, blood pressure, SPO_2_ and body temperature values were significantly lower after anesthesia for the rabbits in the anesthesia group. Thus, we could potentially conclude that the rabbits remain closer to their normal physiological status when the fixator is used than when anesthesia is administered.

Pentobarbital has been extensively used for rabbit anesthesia. Similar to other anesthetic agents, it depresses the cardiac, sympathetic, and adrenal medullary components of the autonomic nervous system. It can also attenuate reflexes involving the sympathetic and parasympathetic nervous systems, and anesthetic agent-induced depression of vascular responses should not be ignored^5,6^. Additionally, complications such as anesthetic death, unsuccessful induction, and premature recovery are common. One of the major problems observed in rabbits is respiratory depression, which may be dangerous for the animal and could result in failure of the experiment^22^.

In rabbits, various anesthetics have differing effects on biochemical indicators and even organ histology^7^, and variations in the tolerance of individual rabbits to anesthetics will result in different anesthetic effects among experimental rabbits^23^. Matchett and Wood^24^ also found that anesthesia affected heart rate variability, which further limits the use of anesthetics in animal experiments. Interestingly, these problems may not have arisen for the rabbits in the fixator group. Changes in stress hormones may influence the pharmacokinetics studies of PET. Specifically, adrenaline can increase glucose utilization rates in hindlimb muscles, skin, ileum, liver, spleen, lung, epididymal fat, and kidney^25^, and cortisol influences the metabolic activity in specific brain regions^26^. Our results showed that adrenaline and cortisol did not significantly change after fixation, which means this fixator is suitable for animal PET researches.

The greatest concern regarding the use of rabbits for imaging studies is artifacts generated by the movement of the animals during the imaging examinations, which adversely impact the image quality and research results.

Drucke et al.^27^ used thermoplastic masks as a noninvasive mold to restrain a primate head and to enable a wide range of neuroscientific experiments. They indicated that these masks allowed for the acquisition of clearer MRI images. In the current study, the imaging quality was compared between the two groups for both anatomical and metabolic imaging. In the anatomical imaging, the images of the rabbits from both groups had similar visual effects, and neither of them had significant motion artifacts.

Based on the analysis of image artifacts using subjective image quality in the main anatomic region^14–17^, the subjective image quality scores for the CT, MRI and PET images of each important anatomic region did not differ significantly between the two groups (Tables 3), and no artifacts that significantly impacted image reading were detected. These results demonstrated that for rabbits, similar quality anatomical imaging was obtained both after anesthesia and after fixation. In PET metabolic imaging, similar visual effects were observed in the images of the rabbits from both groups, and significant motion artifacts were not observed for either group (Table 2, Fig. 3). Overall, the quality of the metabolic imaging after fixation is close to that observed after anesthesia in rabbits.

Furthermore, to evaluate the differences in the scanner performance with and without the thermoplastic mask, phantom studies with different concentrations of iodinated contrast agent, different radioactivity values of ^18^F-FDG and different concentrations of Gd-DTPA were performed. The quantitative analysis showed that the novel fixator made of a thermoplastic mask did not affect the accuracy of the measurements results of CT, MRI and PET. The phantom studies indicate that the thermoplastic mask does not influence the detection of X-rays and gamma photons, nor does it affect the magnetic field of MRI. Thus, precise data for rabbit experiments can be obtained with multimodality image techniques by using this novel fixator.

The imaging examinations of rabbits after anesthesia cannot fully reflect these animals’ physiological status under actual conditions. Anesthetic agents will affect the absorption, distribution, metabolism, and elimination of intravenously and orally administered drugs, which further impact the pharmacokinetics^28^.

The functional imaging performance depends on the scanner model, acquisition parameters, image reconstruction, and patient status^29^. Kersemans et al.^30^ studied the effects of both anesthetic and carrier gas upon the uptake of ^64^Cu-CuATSM, ^99m^Tc-HL91, and ^18^F-FMISO in a preclinical model of tumor hypoxia on PET and SPECT. Their results showed that the use of anesthesia can have profound effects on the experimental outcome. All the tested anesthetics reduced the tumor-hypoxia uptake. The anesthesia also substantially influenced the radiotracer biodistribution and tumor uptake. The studies by Flores et al. and Lee et al. showed that both gas and injected anesthetic agents will affect the uptake of ^18^F-FDG by normal tissue or tumor tissue; i.e., it elevates the blood ^18^F-FDG activity and reduces the tumor uptake ratio through inhibition of insulin release^31,32^.

In the current study, the SUV values of the examined main organs, with the exception of the muscles, were lower in the fixator group than in the anesthesia group. SUV values of liver between two groups were almost identical (Fig. 2). This finding demonstrated that relative to anesthesia, fixation did not have an obvious impact on the organ metabolism. Notably, a large between-group difference was detected for muscle (Fig. 2), which may have reflected the muscular relaxation induced by anesthesia. The SUV values is a relative uptake measure normalized for body weight and injected dose while the dose was distributed over the entire body^33^. ^18^F-FDG uptake of muscle in the anesthesia group was less than that in the fixator group, while the ^18^F-FDG uptake of other organs (brain, lung and kidney) was higher than that in fixator group. We also found that the SUV values stayed constant in the first 30–45 min but slightly decreased over time. This phenomenon may be attributed to the increased elimination of ^18^F-FDG due to the effect of the biological half-life. Normal tissues have a higher content of the glucose-6-phosphatase enzyme; FDG-6-P is dephosphorylated by glucose-6-phosphatase, which then effluxes from intracellular compartment to the extracellular compartment and is excreted through urine, leading to a consequent and gradual decline in FDG-6-P accumulation over time^34,35^. Therefore, in the context of metabolic imaging, the novel fixator might allow for the acquisition of pharmacokinetic information in a relatively normal physiological state.

When carrying out pharmacokinetic studies using imaging techniques such as PET, it is often necessary to acquire different temporal images and measure corresponding values, which requires restraining experimental animals for a long time. The fixator shown in our study can meet this requirement and prevent physiological changes or even deaths of rabbits caused by multiple anesthesia administrations.

Additionally, the acquisition of some images requires the rabbit to maintain some special position for a long time and may require a complicated device and image acquisition apparatus specific to small animals^36^. Anesthetized rabbits are unable to be placed in certain special positions. In our study, the rabbits in the anesthesia group were treated with an additional 1 ml of 3% pentobarbital sodium in 60 min because their muscle tension had recovered. However, once the rabbits were restrained using the thermoplastic fixator, they could be held in the same posture until completion of the examination. On the other hand, the thermoplastic masks can be heat molded as needed before the relevant examinations so that the rabbit can be kept at the special position for a long time for scanning.

We have used the thermoplastic masks for rabbit experiments many times in our research. Because of the extremely high applicability and portability of the thermoplastic masks, a rabbit can often be restrained by two experienced persons, which saves much time and manpower, without using anesthesia.

Our study has some limitations. The sample size for both groups was small, and there may have been selective errors in the measurements. Second, this study used medium-sized experimental rabbits weighing 2100–3000 g to make the fixator, which limited our rabbits to a certain weight range. The fixator would need to be remolded for smaller or larger rabbits. Finally, further study is needed to verify the feasibility of the described fixator-based approach for restraining other animals and conducting assessments other than imaging studies.

## Conclusions

In summary, the novel rabbit fixator allows for the maintenance of a fixed posture for a long period without affecting the rabbit’s heart rate, respiration frequency, blood pressure, or stress hormones; thus, the use of this fixator is highly convenient for image acquisition. Furthermore, fixator use is suitable for multiple imaging modalities in an awake rabbit, has less of an impact on the rabbit’s metabolism than anesthesia, and does not significantly affect the imaging quality.

### Ethical approval

This study was approved by the ethics committee for experimental animals of Kunming Medical University, number KMMU2019091.

## Supplementary information

Supplementary information 1.

Supplementary information 2.
