# Supplementary material for: A novel rabbit fixator made of a thermoplastic mask for awake imaging experiments
Source: Sci Rep. 2021 Jan 15;11:1546. doi: 10.1038/s41598-021-81358-6 (PMC7810717; doi:10.1038/s41598-021-81358-6)
Supplement: Supplementary file 1 — Supplementary information 1. [file 41598_2021_81358_MOESM1_ESM.pdf]

A novel rabbit fixator made of a thermoplastic mask for awake imaging experiments

Rencai Lu[1], Li Hou[2], Siyu Wang[1], Bo She[1], Hong He[1], Wentao Gao[1], Sidang Wang[1],Dongdong Xv[1], Yunhai Ji[1], Shasha Yang[1], Zhaohui Yang[3], Shaobo Wang[1]**

1 PET-CT Center, the First People's Hospital of Yunnan Province, Kunming 650032, Yunnan Province, China.

2 Department of radiation, the First People's Hospital of Yunnan Province, Kunming 650032, Yunnan Province, China.

3 Yunnan Key Laboratory of Primate Biomedical Research, Institute of Primate Translational Medicine, Kunming University of Science and Technology, Kunming 650093, Yunnan Province, China

These authors contributed equally: Rencai Lu and Li Hou.

** Corresponding author:*

Shaobo Wang wshbo_98@126.com

Bo She 1471265639@qq.com

附件 3：

昆明医科大学动物实验伦理审查申请表 编 号

The Applocation For Animal Exprimental Ethical Inspection （No.）: KMMU2019091

<table>
<tr><td rowspan="12">申请人填写的相关信息
(Concerned information wrote by applicant)</td><td colspan="3">申请部门(Name of organization)：云南省第一人民医院(the First People's Hospital of Yunnan Province)</td></tr>
<tr><td colspan="3">项目负责人(applicant)：
王绍波 Shaobo Wang
技术职称(Professional title)：
副教授 Associate Professor
岗位证书编号
(Number of permit): -</td></tr>
<tr><td colspan="3">项目执行人 1(operator)：
鲁仁财 Rencai Lu
技术职称(Professional title)：
住院医师 Resident Doctor
岗位证书编号
(Number of permit): LA2015155</td></tr>
<tr><td colspan="3">项目执行人 2(operator)：
王思宇 Siyu Wang
技术职称(Professional title)：
住院医师 Resident Doctor
岗位证书编号
(Number of permit): -</td></tr>
<tr><td colspan="3">项目执行人 3(operator)：
何宏 Hong He
技术职称(Professional title) ：
住院医师 Resident Doctor
岗位证书编号
(Number of permit): -</td></tr>
<tr><td colspan="3">项目执行人 4(operator)：
朱迪 Di Zhu
技术职称(Professional title)：
住院医 Resident Doctor
岗位证书编号
(Number of permit): LA2017093</td></tr>
<tr><td colspan="3">动物实验环境设施许可证号(Number of Permit): SYXK（滇）K2015-0002</td></tr>
<tr><td colspan="3">实验名称(Name of experiment)：一种用于影像学检查的家兔体位固定器(**A novel rabbit fixator made of a thermoplastic mask for awake imaging experiments)**</td></tr>
<tr><td colspan="3">实验目的(Aim of experiment):
This study aimed to develop and validate a novel rabbit fixator made from a thermoplastic mask for awake imaging experiments.</td></tr>
<tr><td rowspan="3">拟进动物情况</td><td colspan="2">动物来源(Source of animal):昆明医科大学实验动物部
Department of Experimental Animals, Kunming Medical University</td></tr>
<tr><td colspan="2">品种品系(Species or strain)：兔(Rabbit) 等级(Grade): 普通级(conventional) 规格(Specifications):2.0~3.0kg</td></tr>
<tr><td>数量(Number): 20 (♀20； ♂ 0)</td><td>申请日期(Application date): November 1, 2019</td></tr>
</table>

<table>
<tr><td rowspan="5">申请人填写的相关信息</td><td>进驻日期(Entering date):
November 1, 2019</td><td colspan="3">结束日期(Ending date):
August 30, 2020</td></tr>
<tr><td colspan="4">实验要点，包括实验方法、观测指标、实验结束处死动物的方法等(Outline of experiments, experimental methods, observational index, executing animal method et. al):
A novel rabbit fixator was formed and twenty rabbits were randomly divided into fixator (n=10) and anesthesia (n=10) groups. The animals' vital signs, stress hormones (cortisol and adrenaline), and subjective image quality scores for the computed tomography (CT), positron emission tomography (PET), and magnetic resonance imaging (MRI) scanning were measured and compared. After the experiment, rabbits were killed by intravenous injection of air through the marginal ear vessels.</td></tr>
<tr><td colspan="4">声明：我将自觉遵守实验动物福利伦理原则，随时接受委员会的监督与检查，如违反规定，自愿接受处罚(I promise to obey the laboratory animal welfare and ethic principle, and accept the inspection of the committee, otherwise I,ll be punished).
声明人签名(Signature): [illegible] (Shaobo Wang)
Date: November 1, 2019</td></tr>
<tr><td colspan="4">申请人签名(Signature of applicant): Shaobo Wang 联系电话(Telephone):15812082912</td></tr>
<tr><td colspan="4"></td></tr>
<tr><td>审查依据
(Inspection contents)</td><td colspan="4">1. 该项目是否必须用实验动物进行实验，即能否用计算机模拟、细胞培养等非生命方法替代动物或用低等动物替代高等动物进行实验(Does laboratory animal must be used in the project? Could other methods such as computer simulation, cell cultivation or using the low-grade animal instead of the high-grade animal)?
2. 表中所填申请人资格和所用动物的品种品系、质量等级、规格是否合适，能否通过改良设计方案或用高质量的动物来减少所用动物的数量(Are the qualification of applicant, species or strain, grade and specifications of animals suitable? Could the quantity of animals be reduced by improving the study design or using high quality animals)?
3. 能否投通过改进实验方法、调整实验观测指标、改良处死动物的方法，来优化实验方案、善待动物(Could the study design and animal treatment be refined by ameliorating experimental method, adjusting observational index, executing animal method)?</td></tr>
<tr><td>审查结果(是否同意申请人的实验方案)(Results of inspection)</td><td>伦理委员会意见
(Animal Experimental Ethical Committee)</td><td>同意
(Agree)
☑</td><td>不同意
(Disagree)
☐</td><td>签名
(Signature)
签章(Stamp)</td></tr>
<tr><td colspan="4">备注(Supplement):</td><td></td></tr>
</table>

附件 4：

昆明医科大学实验动物设施使用证明

The Certification of Using the housing facility of Laboratory Animal

<table>
<tr><td colspan="6">使用单位(Name of organization):云南省第一人民医院</td></tr>
<tr><td colspan="6">实验名称(Name of experiment): 一种用于影像学检查的家兔体位固定器（A novel rabbit restraint fixator for Imaging experiments using thermoplastic masks）</td></tr>
<tr><td colspan="3"></td><td colspan="3">使用者签名(Signature of user)</td></tr>
<tr><td rowspan="4">使用动物情况
(Situation of using laboratory animal)</td><td colspan="2">来源(Source):昆明医科大学实验动物部</td><td colspan="3">质量者合格证书编号(Number of qualitative qualification):</td></tr>
<tr><td colspan="2">品种品系(Species or strain):实验家兔 rabbit</td><td colspan="3">等级(Grade):普通级</td></tr>
<tr><td colspan="2">数量(Quantity):20只(♀20只； ♂ 0 只)</td><td colspan="3">规格(Specifications):2.0~2.5kg</td></tr>
<tr><td colspan="2">进驻日期(Entering date):
November 1, 2019</td><td colspan="3">结束日期(Ending date):
August 30, 2020</td></tr>
<tr><td rowspan="4">饲养设施条件
(Condition of the housing facilities)</td><td>屏障设施
(barrier facility)
☐</td><td colspan="4">本设施的环境条件符合中国国家标准《实验动物 环境及设施》（GB14925-）对屏障动物实验设施的有关标准，动物饲养管理和动物实验操作符合《云南省实验动物管理条例》等法规的要求(This housing facility is a barrier housing facility, and it has in keeping with national standard《Laboratory Animal-Requirements of Environment and Housing Facilities》(GB 14925-). The care of laboratory animal and the animal experimental operation have conforming to 《Yunnan Administration Rule of Laboratory Animal》, et al)。</td></tr>
<tr><td>普通设施
(Ordinary facility)
☑</td><td colspan="4">本设施的环境条件符合中国国家标准《实验动物 环境及设施》(GB14925-)对普通动物实验设施的有关标准，动物饲养管理和动物实验操作符合《云南省实验动物管理条例》等法规的要求(This housing facility is an ordinary housing facility, and it has in keeping with national standard 《Laboratory Animal-Requirements of Environment and Housing Facilities》(GB 14925-) . The care of laboratory animal and the animal experimental operation have conforming to 《Yunnan Administration Rule of Laboratory Animal》, et al)。</td></tr>
<tr><td rowspan="2">设施使用许可
(Permit of the facility)</td><td>许可证编号：
(Number of permit)</td><td>SYXK（滇）K2015-0002</td><td>有效期：
(Tern of validity)</td><td>自 2015 年 05 月
至 2020 年 05 月</td></tr>
<tr><td>许可证发放机构
(Permitting organization)</td><td colspan="3">☐云南省科技厅 ☑其他 昆明市科学技术局
(Yunnan Province Municipal Committee of Science and Technology)</td></tr>
<tr><td colspan="2">实验设施负责人意见
(Head of the project):</td><td>☑情况属实
(Approval)</td><td colspan="2">☐情况不属实
(Disapproval)</td><td>签 名
(Signature)</td></tr>
<tr><td colspan="5">备注(Supplement):</td><td>签章(Stamp)</td></tr>
</table>

Patent information:

A patent has been authorized by the National Intellectual Property Administration of China under number ZL 2019 2 0133870.4. Website: http://epub.sipo.gov.cn/overTran.action?strWord=%E7%94%B3%E8%AF%B7%E5%8F%B7%3D%272019201338704%27&numType=&numSortMethod=4&strLicenseCode=&selected=&numFM=0&numXX=0&numWG=0&pageSize=10&pageNow=1.
